# Assessment of cardiovascular disease in immune thrombotic thrombocytopenic purpura

**DOI:** 10.1016/j.rpth.2026.106631

**Published:** 2026-05-08

**Authors:** Paschalis Evangelidis, Nikolaos Kotsiou, Panagiotis Kalmoukos, Eleni Gavriilaki

**Affiliations:** 1 Second Propedeutic Department of Internal Medicine, Hippocration Hospital, Aristotle University of Thessaloniki, Thessaloniki, Greece; 2 Endothelial Injury Excellence Centre, Hematology Department, Bone Marrow Transplantation Unit, G. Papanicolaou General Hospital of Thessaloniki, Thessaloniki, Greece

Dear Editor,

We are grateful for the important contribution by Javed et al. [1] entitled “Standard cardiovascular risk prediction scores underestimate risk in immune-mediated thrombotic thrombocytopenic purpura survivors.” The authors presented a very well-organized work, including a large cohort of immune thrombotic thrombocytopenic purpura (iTTP) survivors, aiming to investigate the potential role of well-established cardiovascular risk models, such as 2008 Framingham Heart Study global cardiovascular disease (CVD) and the American College of Cardiology (ACC)/American Heart Association (AHA) atherosclerotic CVD scores, in major adverse cardiovascular events (MACE) prediction. With a median follow-up period of 3.8 years, MACE occurred in 51 (37.8%) patients. In their detailed analysis, Framingham Heart Study and ACC/AHA atherosclerotic CVD estimators were characterized by poor discrimination and calibration, while also showing suboptimal sensitivity and specificity in the prediction of MACE during remission. The authors highlight the possible involvement of other nontraditional factors in the CVD risk of iTTP survivors and the importance of novel risk predictive models personalized for this patient population.

### Cardiovascular risk in iTTP

Indeed, the prevalence of MACE, especially of acute ischemic stroke, is higher in iTTP survivors than in the general population of the same age and gender [2]. This might be attributed to persistent reduced ADAMTS-13 activity, which results in accumulation of ultralarge von Willebrand polymers, contributing to endothelial injury, complement activation, and vascular inflammation, similar to congenital thrombotic thrombocytopenic purpura and sickle cell disease [3]. Based on this hypothesis, early vascular aging, increased arterial stiffness, and microvascular dysfunction potentially can have a crucial role in MACE pathogenesis in iTTP survivors [4]. Thus, the above-described risk estimators, which do not incorporate the aforementioned factors, underestimate the MACE risk in this patient population, as shown in this study [1].

## Noninterventional methods for the evaluation of macrocirculation and microcirculation dysfunction: predicting CVD and mortality

Macrovascular and microvascular dysfunction has been shown in several cohort studies as a predictor of MACE and CVD mortality in both general population and high-risk groups (hypertension and chronic kidney disease) [5]. Stiffening of large arteries, a reliable marker of vascular aging, has been recognized as an established risk factor of CVD and survival in several clinical conditions. Pulse wave velocity (PWV) is the gold standard method for arterial stiffness evaluation. Moreover, early atherosclerosis might have a possible association with MACE development in iTTP during remission. Carotid intima-media thickness (cIMT), easily evaluated by ultrasonography, is considered indicative of systemic atherosclerosis burden [5]. The role of PWV and cIMT in the prediction of MACE in iTTP survivors has to be assessed, given its proven value in several clinical entities, including autoimmune disorders [6].

Alterations in microvascular function are also implicated in the development of CVD. Especially, moderately increased albuminuria (urinary albumin excretion: 30-300 mg/24 or urinary albumin/creatinine ratio: 30-300 mg/g), a recognized noninvasive marker of renal microcirculation, has been shown in several prospective studies as a powerful and reliable estimator of CVD events and mortality [5]. Impairment of myocardial perfusion, which may lead to the development of acute myocardial infarction, can be assessed by subendocardial viability ratio, also known as the Buckberg index, which can be easily calculated using noninvasive techniques [7]. Indeed, subendocardial viability ratio has emerged as a potential predictor of fatal and nonfatal CVD events [7].

Additionally, alterations in retinal vessels have been found as independent predictors of MACE and poor survival even in the general population. These changes can be assessed by noninvasive methods, such as the quantitative evaluation of the retinal vasculature with digital retinal photography. Skin constitutes an easily approachable vascular bed for the evaluation of microvascular dysfunction. While several methods have been used for this aim, laser speckle contrast imaging is one of the robust means of skin microcirculation assessment [8]. Nevertheless, the role of laser speckle contrast imaging in the prediction of MACE has not been validated.

MACE development in iTTP survivors is multifactorial: comorbidities, such as hypertension, chronic inflammation, low ADAMTS-13 remission activity, and treatment-related factors, are implicated [2]. These factors might contribute to macrovascular (increased PWV and cIMT) and microvascular (impaired renal, retinal, myocardial, and skin microcirculation) dysfunction, which have not been studied in iTTP yet. We consider it essential to evaluate the above-described factors at baseline and prospectively examine their predictive role for long-term MACE development, as has been shown in several other clinical entities. Furthermore, the findings from these patients should be compared with normal controls, aiming to better understand the particularities of vascular deformities in iTTP survivors. Multicenter collaboration is crucial in this field, given the rarity of iTTP. In the Figure, we provide an overview of noninvasive techniques for the evaluation of macrocirculation and microcirculation.

**Figure:**
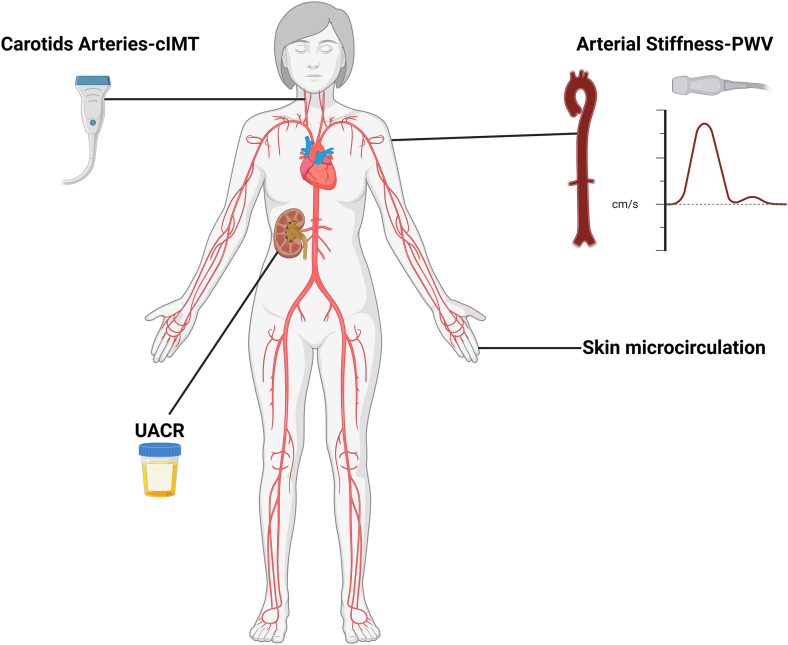
An overview of noninvasive techniques for the evaluation of macrocirculation and microcirculation. cIMT, carotid intima-media thickness; PWV, pulse wave velocity; UACR, urine albumin creatinine ratio. Created in BioRender. Evangelidis P. (2025). https://BioRender.com/x2euq58.
